# Platelet-Rich Plasma Combined with Hyaluronic Acid versus Leucocyte and Platelet-Rich Plasma in the Conservative Treatment of Knee Osteoarthritis. A Retrospective Study

**DOI:** 10.3390/medicina57030232

**Published:** 2021-03-03

**Authors:** Michelangelo Palco, Domenico Fenga, Giorgio Carmelo Basile, Paolo Rizzo, Bruno Cavalieri, Danilo Leonetti, Angelo Alito, Antongiulio Bruschetta, Francesco Traina

**Affiliations:** 1 Department of Biomedical, Dental and Morphological and Functional Images, Section of Orthopedics and Traumatology, University of Messina, 98124 Messina, Italy; michelangelo.palco@gmail.com (M.P.); dfenga@gmail.com (D.F.); rizpaolo@gmail.com (P.R.); brunocavalieri@gmail.com (B.C.); dleonetti@gmail.com (D.L.); traina.francesco@gmail.com (F.T.); 2 Department of Biomedical, Dental and Morphological and Functional Images, University of Messina, 98124 Messina, Italy; 3 U.O.C of Physical and Rehabilitation Medicine and Sports Medicine, Policlinico Universitario G. Martino, 98124 Messina, Italy; alitomedical@gmail.com; 4 Orthopaedic Institute of Southern Italy “Franco Scalabrino”, 98015 Messina, Italy; antongiulio.bruschetta89@gmail.com; 5 Orthopaedic-Traumatology and Prosthetic Surgery and Revisions of Hip and Knee Implants, IRCCS Istituto Ortopedico Rizzoli, Via G.C. Pupilli 1, 40136 Bologna, Italy

**Keywords:** knee osteoarthritis, intra-articular injection, platelet rich plasma, hyaluronic acid, pain, osteoarthritis, cartilage

## Abstract

*Background and objectives*: Knee osteoarthritis (KO) is one of the most common joint diseases, determining knee pain and reduction of mobility, with a negative effect on quality of life. Intra-articular injections of different formulations of platelet-rich plasma (PRP) are an increasingly common non-surgical treatment for KO. Recently, in order to combine the anti-inflammatory effect of platelet rich plasma and the viscosupplementation effect of hyaluronic acid, a formulation of PRP combined with hyaluronic acid (PRP + HA) has been proposed. The purpose of this study is to retrospectively compare the effectiveness of plasma with high concentration of platelets and leukocytes (L-PRP) with PRP + HA in patients with mild to moderate (Kellgren–Lawrence scale II-III grade) KO. *Materials and Methods*: Among the 51 patients included, 28 have been treated with L-PRP, while 23 with PRP + HA. A retrospective evaluation at baseline (T0), after 3 months (T1) and 1 year (T2) has been performed. The outcome analyzed are the Knee Society Score (KSS), the Visuo Analogic Scale (VAS) (at T0, T1, and T2) and the Knee Injury and Osteoarthritis Outcome Score (KOOS) (T0 and T2). We evaluated change in mean scores within and between groups among different time points using repeated measures ANCOVA. *Results*: Although the two treatments have been both effective in reducing VAS, the group treated with PRP + HA showed a significantly lower KSS. *Conclusions*: Our results show that the use of both treatments may help to reduce pain in patients with mild to moderate KO. PRP + HA showed better results in improving knee mobility and function. These results should be considered only preliminary: Further research is needed to completely describe the clinical effectiveness of these formulations.

## 1. Introduction

Knee osteoarthritis (KO) is a common degenerative joint disease, affecting approximately 250 million people worldwide; it often determines knee pain, which limits activity and impairs quality of life [1], determining a risk of mobility disability (defined as the need for help with walking or climbing stairs) greater than in any other medical condition in people ≥65 years of age [2]. Both surgical and non-surgical options. To date, there is no treatment able to prevent or arrest cartilage degeneration: non-surgical options, based on patients education, rehabilitation, and pharmacotherapy, can be administered to reduce swelling, pain, and disability [3], their main aim being to modify the lifestyle, achieve pain control, delay disease progression, and improve function [4,5]. Among these treatments, intra-articular infiltrations with hyaluronic acid (HA) or blood derivatives are employed to achieve pain control and restore knee function [3]. Specifically, the presence of HA in the osteoarthritic joint is thought to have a beneficial effect: the introduction of eterologous HA in the arthritic joint is called viscosupplementation, and is considered crucial to restore the mechanical properties of synovial fluid, thus determining an analgesic, anti-inflammatory, and condroprotective effect [6]. The rationale behind the use of blood derivatives is the ability to provide bioactive molecules which could positively influence the joint environment, and should promote the regeneration of degenerating tissues: in particular, platelet-rich plasma (PRP) has gained increasing attention due to the pool of growth factors stored in platelet α-granules, that, according to recent studies, could sustain the regeneration of articular cartilage [7,8]. Clinical effectiveness of HA injections, as well as safety, has been proven by several studies [9]: in patients with mild-moderate KO, PRP administration shows comparable effectiveness to steroid/anesthetic or HA [10]. Furthermore, the use of PRP could be appropriate not only in the treatment of mild-moderate KO: also patients with severe KO not eligible for knee replacement (due to pre-existing conditions or other personal reasons) could benefit from PRP injections [10]. The few available randomized controlled trials give overall support to PRP injections for knee OA treatment [8].

Several different formulations of PRP can be employed in the management of KO. Recently, in vitro studies compared leukocyte-poor PRP with leukocyte and platelet-rich plasma (L-PRP) on human chondrocytes [11]. Leukocyte-poor PRP was shown to attenuate the in vitro production of inflammatory cytockines, and to conversely reduce the chondrocytic production of HA [11]. Hence, in order to combine the anti-inflammatory effect of PRP and the viscosupplementation effect of HA, the idea of combining PRP and HA was recently proposed [12].

Several clinical reports have shown that tissue regeneration could be promoted by the use of a PRP + HA formulation. PRP + HA may be effective to improve mobility, relieve pain, and reduce the risk of infections. Nevertheless, the molecular mechanism leading to tissue regeneration has been not completely described. Furthermore, clinical studies on this topic are often based on a restricted number of patients. Therefore, evidence supporting the effectiveness of this formulation is still considered limited and controversial [12,13].

Additionally, to the best of our knowledge, there is no clinical evidence comparing pain perception and objective functional outcomes of PRP + HA with L-PRP in patients with low to mild-moderate grade of KO.

Hence, in this retrospective study, we reviewed the outcomes of knee intra-articular injective therapy administered to outpatients treated in our structure between 2017 and 2018. The aim of this study was to report the effects of PRP + HA and L-PRP intra-articular injective treatment on pain and knee function. Finally, we compared the outcomes to determine whether one of the two treatments gave better clinical results compared to the other one.

## 2. Materials and Methods

### 2.1. Patient Selection

This study was conducted at the Policlinic “Gaetano Martino” (Messina, Italy) and authorized by the local ethical committee (registration: Comitato Etico di Messina, N°0014580). This was designed as a retrospective observational study, aiming to find differences in the outcomes between patients treated with PRP + HA and L-PRP.

Participants have been selected among the patients suffering from KO, treated in our facility between December 2017 and December 2018. Standard follow-up for patients treated with injective therapy lasted one year: therefore, follow-up of the last patient included in the study ended in the month of December 2019.

In all the patients, KO was diagnosed and classified by clinical evaluation and anteroposterior radiograph of the osteoarthritic knee. Radiographic appearance of the most involved knee was evaluated basing on the Kellgren–Lawrence staging system (KL) [14]. According to KL, KO can be doubtful (grade I); mild (grade II); moderate (grade III); severe (grade IV); the radiological evaluation of the patients was performed by 2 experienced observers with several years of work in the orthopedic field (M.P.-P.R.).

In case of doubt, the senior author (F.T.) was available to contribute to the evaluation.

Injective treatment was indicated in patients suffering from KO, reporting knee pain without relief with anti-inflammatory agents, with normal blood and coagulation profile (platelets between 150,000 and 450,000/mm^3^).

We considered patients not eligible to injective treatment if they presented hematological or oncological diseases.

Due to the influence that any of the following conditions may have on the outcomes in exam, patients were excluded from the retrospective evaluation if they presented/reported: knee injuries or trauma, arthritis, moderate genu varus or valgus, bucket-like meniscal lesions, joint infections, bone necrosis, use of corticosteroids in the last three months before evaluation, routine use (e.g., due to comorbidities) of drugs or physical therapy (e.g., cryotherapy) for analgesic/anti-inflammatory purposes, history of spine or lower limb surgery.

Patients not releasing informed consent, not present to one follow-up visit, or (for any reason) undergoing follow-up evaluation at an anticipated or delayed time point (±15 days) were excluded.

To evaluate the effects of the treatment in patients with mild to moderate disease, we decided to exclude from our retrospective revision patients with severe or very low KL grade (grade I and IV).

We estimated sample size using G*Power software. We considered a minimal sample size of 44 subjects (22 per group): we adopted an α level = 0.05, power (1-β) = 0.95, and effect size of 0.25 (medium effect size) [15,16].

All the patients have received one infiltration in the most symptomatic knee every 15 days for a total of 3 injections in 30 days (on the 1st, 15th, and 30th day).

Regardless of the treatment administered, physiotherapy and gradual muscle strengthening were prescribed since the second infiltration of the cycle to all the patients.

### 2.2. Treatment

For each infiltration, a single dose of 8 mL of venous blood from the cubital vein was collected in a sterile test tube. Subsequently, we used RegenKit^®^-THT-3/RegenCell^®^ (REGEN LAB SA, En Budron B2, 1052 Mont-sur-Lausanne, Switzerland) to obtain L-PRP and CellularMatrix A-CP-HA to obtain PRP + HA.

PRP + HA and L-PRP preparation were fully automated processes, not based on operator dependent results.

L-PRP was obtained after centrifugation of venous blood for 9 min at 3400 rpm/1500× *g*; PRP + HA instead, was centrifuged for 5 min at 3400 rpm/1500× *g*. The volume obtained from each individual tube stood at 5 mL (in the case of PRP + HA, 5 mL equals to 3 mL of PRP and 2 mL of HA) [17].

According to the performance tests carried out by the producing company, (according to the U.S. Food and Drugs Administration requirements for PRP medical devices) CellularMatrix A-CP-HA had a platelet concentration factor of 1.6× and a platelet recovery of approximately 80%: the obtained PRP + HA had a mean concentration of 310,000 platelets/mm^3^ and 1000 leukocytes/mm^3^ (in the PRP fraction); RegenTHT had a platelet concentration factor of 1.7×, with a platelet recovery of approximately 95%: the obtained L-PRP reached a mean concentration of 370,000 platelets/mm^3^ and 4000 leukocytes/mm^3^.

Moreover, a functionality test performed in our structure (with the contribution of 10 healthy volunteers, between 25 and 56 years of age) showed an average content of 800 leukocytes/mm^3^ and 290,000 platelets/mm^3^. Mean content for L-PRP was 3600 leukocytes/mm^3^ and 340,000 platelets/mm^3^, consistently with the data provided by the producing company. Cells and platelets concentrations were determined by the use of an automatic cell counter (BC 6800, Auto Hematology Analyzer, Mindray, Shenzhen, China).

### 2.3. Outcome Measures

To assess the efficacy of the treatment on knee function and pain, we evaluated patients using the Knee Society Score (KSS), the Knee injury and Osteoarthritis Outcome Score (KOOS), and the Visuo-Analogic Scale (VAS).

The KSS is based on therapist observation and comprises a knee score and a functional score: the first one evaluates aspects of mobility (range of motion, flexion contracture, extension lag, alignment, and stability in the anteroposterior and mediolateral plane), with a maximum score of 100 points. The functional score is based on walking (50 points), stair climbing (50 points), and on the possible use of walking aids (which can subtract up to 20 points). Scores of 80–100 are empirically considered excellent, 70–79 good, 60–69 fair, and <60 poor [18].

The KOOS is a self-reported questionnaire rating five dimensions: pain, other symptoms, activities of daily living, sport and recreation function, and knee-related quality of life. Total scores from different subscales are translated to a total score ranging from 0 (extreme functional impairment) to 100 (no impairment) [19].

The Visual-Analogue Scale (VAS) was in use for the measurement of intangible quantities (such as pain) since the 1920s. It consists of a line usually 100 mm in length, with anchor descriptors such as (in the pain context) “no pain” and “worst pain imaginable”. The patient marks the line to indicate the pain perception: the outcome is represented by the distance from the left endpoint to the mark, measured in mm [20].

Each patient was evaluated with VAS and KSS before treatment (baseline evaluation: T0), 3 months (T1), and 1 year (T2) after treatment. KOOS was administered only at the start and at the end of the follow-up (T0 and T2). Administration of the therapy was performed in outpatient regimen, as well as the clinical outcomes collection.

### 2.4. Statistical Analysis

We analyzed the differences between the two groups for gender, KL grade (χ^2^ test), and mean age (*t*-test). At baseline, the two groups mean scores of outcome variables (KOOS, KSS, and VAS) were compared using independent-samples *t*-test.

The change in mean KSS, VAS, and KOOS scores at different time points was examined using repeated measures ANCOVA with post hoc pairwise analysis. Dependent variable was the score in exam, while time was the independent variable and age was set as covariate.

To evaluate the effect of different treatments (PRP + HA or L-PRP) in the outcome scores (KSS, VAS, KOOS), we used a general linear model (repeated measures ANCOVA) analyzing differences in the mean values of the outcome variables (KSS, VAS, KOOS) between the levels of a within-subject factor (time), between the levels of a between-subject factor (treatment), and examining the interaction of the two. Age was set as a covariate. Bonferroni correction for multiple comparisons was adopted: thus the significance level was set at *p* = 0.008 (*p* = 0.05/6).

All statistical analysis was performed with SPSS version 25.0 (IBM Corp, Armonk, NY, USA).

## 3. Results

### 3.1. Descriptive Statistics

**Figure 1:**
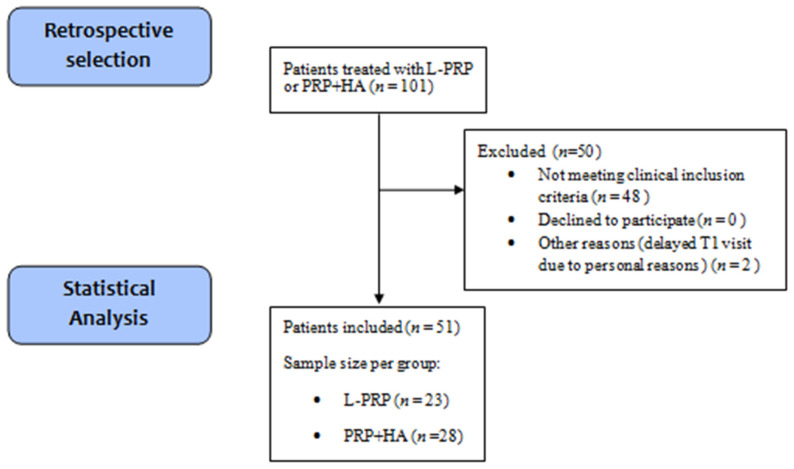
Disposition diagram summarizing the retrospective selection of patients, the number of patients excluded, and the final sample size obtained. *n* = number of subjects. L-PRP = Leukocyte and Platelet-rich plasma, PRP + HA = Platelet-Rich Plasma +Hyaluronic Acid, T1 = Time point 1.

Among 101 patients treated with PRP + HA or L-PRP, 50 did not met the inclusion criteria: 48 patients were excluded due to aforementioned clinical conditions, 2 patients did not respect the date of the 3 months follow-up due to personal reasons, no patient refused informed consent, underwent surgical treatment or interrupted the follow-up. We identified 51 patients suffering from KO, treated with intra-articular injection of one of the two PRP formulations between December 2017 and December 2018, and meeting all the other selection criteria. Among the 51 patients, 23 were treated with L-PRP and 28 with PRP + HA. From this point, patients will be considered as part of two separate groups (L-PRP and PRP + HA). All patients included in the statistical analyisis received the treatment without any notice of harmful/unespected effects. A disposition diagram summarizes patients selection (Figure 1).

At the moment of the injection, out of 51 patients, 18 (35.29%) were suffering from a II KL grade KO, while 33 patients (64.71%) from a III grade. Among the 51 patients, 27 were females (52.94%) and 24 males (47.06%).

Groups did not show differences for gender and KL grade, but patients treated with PRP + HA had a higher mean age (*p* = 0.002). All the baseline characteristics are summarized in Table 1.

#### 3.1.1. Effect of L-PRP and PRP + HA

Both groups showed improvements in mean VAS, KSS, and KOOS at the 1-year post-treatment assessment (reduced VAS, increased KSS and KOOS). Nevertheless, PRP + HA determined significant changes in KSS and VAS, and L-PRP in VAS only: scores, and *p*-values and effect sizes of repeated measures ANCOVA are summarized in Table 2.

In L-PRP group, post-hoc Bonferroni pairwise comparison showed that KSS scores improved significantly only between T0 and T1; in PRP + HA group instead, values significantly improved after every time point (T0–T1 *p* < 0.001; T1–T2 *p* = 0.006). Additionally, post-hoc pairwise comparison of VAS scores in both groups highlighted a significant improvement at every time point (T0–T1 *p* < 0.001; T1–T2 *p* < 0.001).

#### 3.1.2. L-PRP vs. PRP + HA

For KSS, comparison between L-PRP and PRP + HA highlighted a significant main effect of treatment (F(1,48) = 8.247, *p* = 0.006, η^2^ = 0.147), as well as a significant interaction between time and treatment (F(1,48) = 19.055, *p* < 0.001, η^2^ = 0.284).

In particular, pairwise comparison showed a significant simple effect of treatment both at T1(F(1,48) = 10,620 *p* = 0.002, η^2^ = 0.181) and T2 (F(1,48) = 11.745, *p* = 0.001, η^2^ = 0.197), with PRP + HA leading to higher scores (adjusted mean difference: T1 = 5.804; T2 = 8.911).

For VAS and KOOS, no main effect of treatment was highlighted.

**Figure 2:**
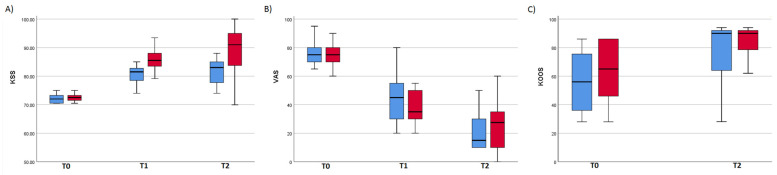
Box plots showing the graphical representation of the values of KSS (**A**), VAS (**B**), and KOOS (**C**) at the different time points of the study. The bottom and the top of each box represent the lower and upper quartiles, respectively, and the line inside each box indicates the median value. Bottom and top bars represent the minimum and maximum value for each group. Analysis on KSS values (**A**) revealed the only main effect of treatment on outcome scores. KSS = Knee Society Score, VAS = Visuo Analogic Scale, KOOS = Knee Injury and Osteoarthritis Outcome Score, T0 = baseline, T1 = Time point 1, T2 = Time point 2.

Figure 2 shows the difference of KSS scores for the two different treatment groups. In KOOS and VAS instead, no significant difference between groups was found (Figure 2).

## 4. Discussion

In this study, clinical outcomes of patients suffering from mild or moderate KO (KL stage II-III), treated with L-PRP or PRP + HA intra-articular injections, were retrospectively compared. Outcomes were observed 3 months and 1 year after the injective treatment.

A significant improvement of pain score (measured with VAS) was present in both groups. Pain reduction was effective after 3 months, and improved after 1 year. The group treated with PRP + HA showed a significant improvement of KSS score, which evaluates knee mobility and function. In both groups the improvement in KOOS, a self-reported measure of functional ability and knee-related quality of life, did not reach significance.

In recent years, properties of different formulations of PRP have been frequently compared: In 2014, for instance, L-PRP was compared with PRP in vitro on human articular chondrocytes. Cavallo and colleagues reported how L-PRP stimulates chondrocytic secrection of HA, which determines a positive effect due to its viscoelastic, analgesic, anti-inflammatory, and chondroprotective properties; in opposition, they suggested that PRP could reduce inflammatory cytokines production [11].

The absence of a strong stimulating effect for the production of HA and the benefits of a viscosupplementation effect led to the idea of combining PRP + HA [21].

Recently, clinical studies have shed new light on the different properties of PRP, HA, and PRP + HA in patients with KO. Examining pain perception and knee function in patients treated with either PRP or HA, Di Martino et al. observed a significant improvement in symptoms, function, and activity which remained stable up to 2 years, regardless of the formulation adopted. The authors observed a pain relieving effect up to 9 months for HA and 1 year for PRP, but these results did not reach significance. Furthermore, a significantly lower rate of re-intervention at 2 years was reported in the PRP group [22]. Our results are consistent with the aforementioned study, by showing an effective and significant 1-year pain reduction regardless of the PRP formulation employed.

In addition, it is worthy to note that in our work, the analgesic effect was still present at the last follow-up. As the VAS scores continuously improved through the 1-year observation, we may speculatively suggest that pain reduction could have persisted months after the last follow-up. In the PRP + HA group, not only VAS, but also KSS showed persistent improvement: specifically pairwise comparison highlighted that KSS of the L-PRP group improved from baseline to T1 (3 months), but remained stable from T1 to T2 (1 year). Otherwise, the PRP + HA group showed significant improvement up to the 1-year follow-up (T2): this result suggests that the while L-PRP effect on knee function and mobility seemed to reach a plateau, remaining stable after T1 (from 3 months to 1 year), PRP + HA effect on the injured joint sustainedly grew over time (still improving after 1 year).

In addition, Lana et al. compared the effects of the administration of PRP + HA, PRP, and HA in patients with mild-moderate KO [23]. In the cited study, the PRP-treated group had a significant reduction in pain (VAS) after 1, 3, 6, and 12 months and a greater improvement in physical function after 12 months; indeed, PRP + HA showed better outcomes in pain and functional limitation when compared to HA alone at 1-year post-treatment and significantly increased physical function at 1 and up to 3 months when compared to PRP alone. The authors described the use of autologous PRP as an effective treatment of mild to moderate knee osteoarthritis, and the use of PRP + HA as a treatment of choice, due to better results after 1 and 3 months [23]. We would substantially agree to consider both PRP and PRP + HA effective in KO and to prefer the use of PRP + HA due to patient’s higher functional improvement. Nevertheless, in our work, PRP + HA treatment yielded more pronounced results, determining a significant improvement in knee function (KSS) not only after 3 months, but also after 1 year. It is worthy to note that while Lana et al. used only patient-reported outcomes, in our work, we administered KSS (depending on objective scores), which was the only outcome measure to differ between groups. The use of different measures could possibly explain the discrepancy between the outcomes: even the authors suggested that the use of only self-reported questionnaires could limit the objectivity of the results [13,23]. Similarly, in our study, no significant effect of treatment was observed in patient-reported measures: specifically, KOOS improvement did not reach significance.

An important element, which may possibly affect the outcome of injective treatment, is the stage of disease of the patients: while most of the studies regarding conservative therapy in KO include mainly patients in early stages, grade III and IV are less represented. Meheux and colleagues reported that the worst results are achieved for K-L grade III to IV [23]. The aim of the current study was to compare the two PRP formulations only in patients with KL II-III, but could be relevant to describe the effects of these treatment specifically in patients with severe KO (IV KL grade). In our opinion, clinical experience suggests that both L-PRP and PRP + HA could induce improved function, mobility, and pain relief also in patients with severe KO; nevertheless these are only suppositions: further evidence is needed to understand whether, and to what extent, different PRP formulations could actually improve clinical conditions of patients with severe KO.

This study presents several limitations: due to the retrospective nature of the study, the baseline scores in exam derives from a single measurement on a nonrandom sample: In this work, both design and analysis did not account for the regression to the means: therefore the results should be only considered preliminary.

We treated patients who did not respond to analgesic/anti-inflammatory therapy, and we interviewed patients about their drugs consumption (to exclude routine analgesic/anti-inflammatory drugs users); nevertheless, we did not systematically collect concomitant treatment. Therefore, we cannot report the occasional consumption of analgesic/anti-inflammatory drugs (e.g., FANS) from the patients.

Another limitation to this study is the absence of follow-up after 1-year: At this time point, the benefits of treatment were still observable, but we were not able to determine how much they persisted, or if the outcomes lasted longer in one group or in the other.

Herein, a formulation of PRP + HA is compared with L-PRP: Due to the retrospective nature of the study, we had not the possibility to compare the effects with placebo, with saline, or especially with other combinations (e.g., PRP only, or L-PRP + HA).

Finally, despite partly accounting for the possible selection bias by adjusting for age, a proper analysis based on matching, if feasible (e.g., prospective setting) may have been superior.

## 5. Conclusions

To sum up, we observed a significant effect of the two formulations in exam in reducing knee pain: The benefit persisted up to 3 months and 1 year after the infiltrative therapy.

Furthermore, PRP + HA determined a significant improvement on knee mobility and function, with a constant improvement which prolonged its effect up to the one year follow up. The group treated with intra-articular injection of PRP enriched with HA showed a higher improvement in functional outcome and knee mobility, but similar results in terms of pain and self-reported level of activity.

In our point of view, we encourage the use of PRP injections as a simple, safe, and minimally invasive treatment approach. Our comparison of PRP + HA and L-PRP suggests that the first could possibly determine better functional and mobility outcomes. However, our work could not be sufficient to definitively suggest a therapeutic choice: Further evidence is needed to identify whether or not one of the two formulations could be more indicated.

Future studies could better describe the use and the efficacy of these biological treatment, comparing them to other non-surgical options or analyzing their effectiveness in severe OA or in other degenerative joint diseases.

## Figures and Tables

**Table 1:** Demographic data of the patients in study. Age was significantly different between groups. Age is expressed as mean ± standard deviation (M ± SD). *N* = number of patients. L-PRP = Leukocyte and Platelet-rich plasma, PRP + HA = Platelet-Rich Plasma +Hyaluronic Acid.

Treatment Group		L-PRP	PRP + HA	Total
*N*		23	28	51
Age		54.04 ± 10.4	62.71 ± 7.88	58.80 ± 10.00
Sex	M/F	12/11	12/16	24/27
K-L grade	II/III	10/13	8/20	18/33

**Table 2:** VAS, KSS, and KOOS scores of the two groups. VAS = Visuo Analogic Scale, KSS = Knee Society Score, KOOS = Knee Injury and Osteoarthritis Outcome Score. T0 = baseline, T1 = Time point 1, T2 = Time point 2.

	T0	T1	T2	*p*-Value *; (within Groups)	Effect Size *
**VAS (L-PRP)**	**75.65 ± 8.16**	**43.70 ± 14.48**	**22.61 ± 16.01**	***p* = 0.005**	**η^2^ = 0.279**
**VAS (PRP + HA)**	**75.89 ± 8.5**	**38.75 ± 10.68**	**25.18 ± 15.48**	***p* = 0.008**	**η^2^ = 0.170**
KSS (L-PRP)	71.13 ± 3.21	79.60 ± 4.72	81.00 ± 6.64	*p* = 0.135	η^2^ = 0.091
**KSS (PRP + HA)**	**70.91 ± 4.65**	**85.20 ± 6.29**	**88.83 ± 9.58**	***p* = 0.004**	**η^2^ = 0.281**
KOOS (L-PRP)	64.53 ± 22.56	//	83.17 ± 14.28	*p* = 0.270	η^2^ = 0.058
KOOS (PRP + HA)	56.17 ± 21.80	//	79.34 ± 17.74	*p* = 0.681	η^2^ = 0.009

Scores are expressed as mean ± standard deviation (M ± SD). Data from the 51 subjects included in statistical analysis. * Results by repeated measures ANCOVA (adjusted for age). Significant results have been reported in bold characters.
